# BODIPY-Based Molecules, a Platform for Photonic and Solar Cells

**DOI:** 10.3390/molecules26010153

**Published:** 2020-12-31

**Authors:** Benedetta Maria Squeo, Lucia Ganzer, Tersilla Virgili, Mariacecilia Pasini

**Affiliations:** 1 Istituto di Scienze e Tecnologie Chimiche (SCITEC), Consiglio Nazionale delle Ricerche (CNR), Via A. Corti 12, 20133 Milano, Italy; benedetta.squeo@scitec.cnr.it; 2 Istituto di Fotonica e Nanotecnologie (IFN), Consiglio Nazionale delle Ricerche (CNR), Dipartimento di Fisica, Politecnico di Milano, P.zza Leonardo da Vinci 32, 20132 Milano, Italy; lucia.ganzer@polimi.it

**Keywords:** BODIPY, optoelectronics, photonics, solar cells, organic dyes

## Abstract

The 4,4-difluoro-4-bora-3a,4a-diaza-s-indacene (BODIPY)-based molecules have emerged as interesting material for optoelectronic applications. The facile structural modification of BODIPY core provides an opportunity to fine-tune its photophysical and optoelectronic properties thanks to the presence of eight reactive sites which allows for the developing of a large number of functionalized derivatives for various applications. This review will focus on BODIPY application as solid-state active material in solar cells and in photonic devices. It has been divided into two sections dedicated to the two different applications. This review provides a concise and precise description of the experimental results, their interpretation as well as the conclusions that can be drawn. The main current research outcomes are summarized to guide the readers towards the full exploitation of the use of this material in optoelectronic applications.

## 1. Introduction

The rapid appearance of organic optoelectronics as a promising alternative to conventional optoelectronics has been largely achieved through the design and development of new conjugated systems [1]. The foremost advantage of organic materials is that they are cheap, lightweight, and flexible and they can offer the opportunity to make the electronics sector greener through the use of abundant materials and manufacturing methods rely on fewer, safer, and more abundant raw materials and with cheaper processing based on solution techniques so the embodied energy for organic devices is expected to be lower than for their inorganic counterparts usually processed by vacuum and high-temperature techniques [2,3,4,5,6,7].

**Figure 1:**
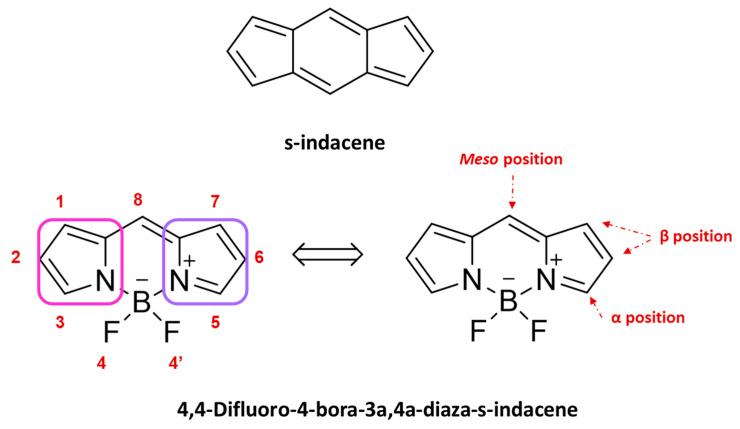
Naming and numbering of the 4,4-difluoro-4-bora-3a,4a-diaza-s-indacene (BODIPY)-based on s-indacene. Pyrrole ring pink boxes and azafulvene violet boxes Reproduced from Ref. [12] with permission of Taylor and Francis.

Since its discovery [8], the 4,4-difluoro-4-bora-3a,4a-diaza-s-indacene (BODIPY) molecule has been the subject of considerable interest thanks to its chemical stability, easy functionalization, and interesting optical and electronic properties combined with low toxicity and high biocompatibility and it has therefore emerged as a promising platform for multiple optoelectronic applications [9,10,11,12]. This type of structure is commonly described as an example of a “rigidified” monomethine cyanine dye or, as it has three p-delocalized rings, one is pyrrole and the others are azafulvene and diazaborinin-type ring systems [13], by analogy with the all-carbon tricyclic ring can be considered as a boradiazaindacene, and the numbering of any substituents follows rule setup for the carbon polycycles (see Figure 1). This dye has also been [14] called “porphyrin’s little sister” and this happy definition has been so successful that in analogy with porphyrinic systems, the 8-position is often referred to as the meso site.

Over the years, several synthetic strategies have been implemented which have allowed the modulation of the optoelectronic properties of the BODIPY that is assumed to have two equivalent resonance forms resulting in the forming of two rings named azafulvene and pyrrole. Pyrrole is aromatic whereas azafulvene is a quinoid-type ring and it is not possible to distinguish the two forms [13]. Depending on the position and type of the substituents, it is possible to modify different parameters such as the delocalization of the π-electrons in the molecule and the supramolecular organization and the morphology of the solid thin film [15,16,17]. In this regard, the BODIPY core has eight reactive positions [18] that can be used to modulate its optical properties [19,20] (Figure 1): the two Boron positions, the two α-positions, the four β-positions, and the meso-position. The positions α and β are those which have the greatest influence on the electronic delocalization, while the other positions, meso and Boron, have a lower impact on electron delocalization but have a strong impact on the steric hindrance of the molecule. The new class of compounds referred to as AZA-BODIPY with a nitrogen substituted to a carbon in meso-position [21,22] will not be discussed in this review.

BODIPY-based materials are playing an increasingly important role in the field of organic semiconductors as demonstrated by the high number of citations about 11,000 achieved from 2019 (Google scholar source) of which 5300 in the year 2020.

This review intends to provide an overview of the recent progress on the design and development of BODIPY-based semiconductor molecules focusing only on molecular materials since polymeric systems require different treatment (discussion) for their application as active materials in solar cells and photonic devices [23,24].

This review is divided into two main parts:

(1) BODIPY-based active materials in OPV. Since numerous comprehensive review articles have focused on the synthetic strategy [25] and general working principles of the organic photovoltaic (OPV) devices, in this review we focus more on the analysis of the best results obtained from the first published data in 2009 [26,27] until the last one in 2020 [28,29].
(2) BODIPY-based active materials in photonics devices. In particular, we focus on the work carried out to optimize the lasing performance and photostability in solid-state, as compared to solutions.

We then conclude with the future perspectives of the use of this promising molecule for photovoltaic and photonic applications. 

## 2. BODIPY-Based Active Materials in OPV

Renewable energy generated by solar cells is one of the potential solutions to the problem of maintaining our energy supply. Organic solar cells have the potential to be part of the next generation of low-cost solar cells. An encouraging state-of-the-art device has exhibited power conversion efficiencies (PCEs) over 17% [30], which represents a crucial step toward the commercialization of OPVs. Among the rapid development of optical-active donor/acceptor materials, interface materials, and device engineering, the properties of donors and acceptors, such as absorption, energy levels, and bandgaps, play a vital role in realizing high PCEs. Therefore, the evolution of new photovoltaic materials with a smaller optical bandgap (E_g_) reasonable highest occupied molecular orbital (HOMO) and lowest unoccupied molecular orbital (LUMO) levels is crucial to further improve the PCEs of OPVs [31]. For these reasons, in the last 10 years BODIPY-based molecules have been of great interest as active materials for photovoltaic devices, thanks to their good absorbance at low energies (low optical gaps), high molar extinction coefficients, good chemical-stability, and opportune redox properties [23]. Table 1 summarizes the results in OPV where a BODIPY-based molecular material has been used as an active layer and the main parameters of a photovoltaic device are mentioned such as the current density J, the fill factor (FF), and the power conversion efficiency (PCE), where the FF is a measure of the quality of the solar cell and it is calculated by comparing the maximum power with the theoretical one [32].

The BODIPY derivatives have good solubility in common organic solvents, suitable HOMO-LUMO levels (see Table 1), and as previously mentioned, possible functionalization at several positions (Figure 1). Despite these interesting features, only recently satisfactory results with PCE above 5%, have been obtained and they are discussed in detail and reported in Table 2. 

**Figure 2:**
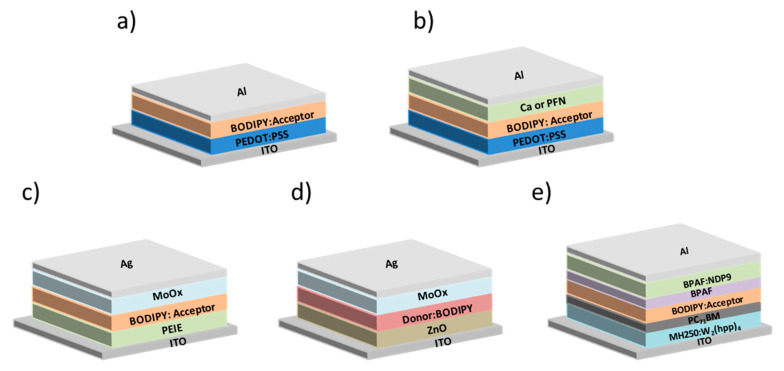
Different Organic Solar Cells (OSCs) architectures are presented in the work. (**a**,**b**) Direct architecture; (**c**,**d**) inverted architecture; (**e**) architecture presented in ref. [42] with an intrinsic layer between n- and p-doped active materials.

Thanks to the planar core, easy lateral functionalization, and electron-deficient characteristics BODIPY derivative is an ideal acceptor unit [33] for the design of donor–acceptor D–A type semiconducting architectures [34,35] which is one of the most used approaches to modulate the optoelectronic properties of organic semiconductors [36,37,38]. The BODIPY derivatives have been used both as donors and acceptors in OPV devices, in direct and inverted architecture [23]. Some of the most interesting results have recently been obtained using conjugated polyelectrolytes at the interface with the metal cathode [29,39]. This class of materials allows the engineering of the interface [40,41] between the metal and the active layer improving the extraction of the charge without complicating the preparation of the device. In fact, for the preparation of films, it is possible to use solution techniques thanks to the use of solvents orthogonal to the active layer. It is important to underline that the parameters that influence the OPV efficiency as mentioned before, are many. It is not always possible to evaluate the individual contribution because they are often intimately connected and closely related to the chemical structure and the choice of position, type, and the number of lateral substituents. In this part of the review on the OPV devices, we report most of these parameters and we try to analyze their individual contribution. All the device architectures have been reported in Figure 2.

### 2.1. BODIPY Molecules as Electron Acceptor Materials in Organic Solar Cells

The fullerene has been, so far, the most common material used as an acceptor in an organic solar cell, however in recent years the search for non-fullerene acceptors has gained significant attention. At the same time, there has also been an increasing effort in the development of small molecules to be used as active materials for OPVs, due to the potential control of frontier orbital energy level that they offer [43]. The combination of these two interests has triggered research for small molecules, non-fullerene acceptors, for bulk heterojunction (BHJ) OPV.

To the best of our knowledge, only two research groups have reported on the synthesis of non-fullerene acceptor based on BODIPY.

**Scheme 1:**
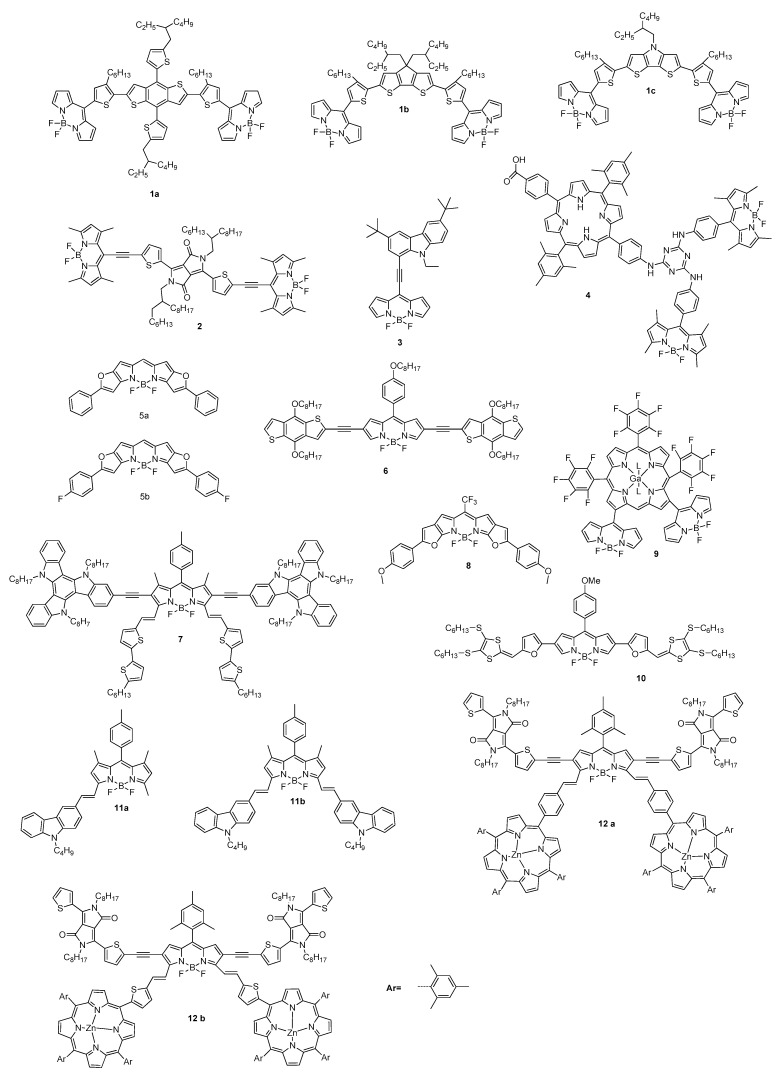
Chemical structure of BODIPY-based molecules used as an acceptor in organic photovoltaic (OPV) devices (**1** and **2**) and as donor **3**–**12**.

In 2014, Thayumanavana et al. [44] presented a series of acceptor–donor–acceptor molecules containing terminal BODIPY unit conjugated through meso-position using a 3-hexylthiophene linker to thiophebenzodithiophene, cyclopentadithiophene, or dithienopyrrole (**1a**, **1b**, **1c**, Scheme 1). The three molecules have deep LUMO energy levels of −3.79 eV, −3.82 eV, and −3.74 eV, and good visible absorption, with absorption maxima in thin-film at 532 nm, 530 nm, and 532 nm, respectively. The molecules have been tested in inverted solar cells with the architecture ITO/ZnO/P3HT:acceptor (40 nm)/MoO3 (7 nm)/Ag (100 nm), with a maximum PCE of 1.51% obtained with the molecules containing cyclopentadithiophene.

In 2017, Zhan et al. [45] firstly reported the synthesis of a new acceptor small molecule based on a diketopyrrole core and two BODIPY lateral moieties (**2**, Scheme 1). The molecule has a LUMO of −3.79 eV and is present in a solid-state, an intense plateau-like absorption with two maxima at 681 nm and 756 nm, which can be attributed to the delocalization of the LUMO along all the molecule.

The small molecule has been tested as an acceptor in direct OSC with architecture glass/ITO/PEDOT:PSS/active layer/Ca/Al, using as donor P3HT, p-DTS-(FBTTh_2_)_2_, and PTB7-Th. The best results have been obtained with P3HT in D/A ratio 1:1.5 with a PCE of 0.888%. To improve the efficiency, the small molecule has been tested also in ternary blend solar cells with p-DTS-(FBTTh_2_)_2_ and P3HT in D1/D2/A ratio 0.5:0.5:1 and a PCE of 2.836% has been obtained. 

### 2.2. BODIPY Molecules as Donor Materials in Organic Solar Cells

In Table 1, the main works on BODIPY-based molecules as a donor material in the OPV devices are reported. For each research work, the year of the research publication, the peak of the absorption and the HOMO and the LUMO position of the new molecule are reported, together with the main parameters of an organic photovoltaic cell. The works are listed in chronological order and we observe that the first solar cell with a BODIPY-based active layer dates back to 2009 with a PCE of 1.7% and that just 6 years later, in 2015, a PCE of around 5% was achieved.

**Table 1:** Photophysical and photovoltaic characteristics of bulk heterojunction organic solar cells based on BODIPY small molecules.

Year	Abs^Film^_Max_ Film [nm]	HOMO [eV]	LUMO [eV]	J_sc_ [mA/cm^2^]	V_oc_ [V]	FF [%]	PCE [%]	Reference
**2009**	572	−5.69	−3.66	4.7	0.866	42	1.7	[27]
**2009**	646	−5.56	−3.75	4.14	0.753	44	1.34	[26]
**2010**	649	-	-	7.00	0.75	38	2.17	[46]
**2011**	733	−4.71	−2.57	6.3	-	67	3.7	[47]
**2012**	580	−5.47	−3.48	8.25	0.988	39.5	3.22	[48]
**2012**	673	−4.26	−3.75	2.9	0.51	35	0.52	[49]
**2012**	714	−5.32	−3.86	14.3	0.7	47	4.70	[50]
**2014**	748	−5.00	−3.59	7	0.68	31	1.50	[51]
**2014**	733	−5.02	−3.64	6.8	0.67	34.3	1.56	[52]
**2014**	760	−5.02	3.37	8.9	0.51	34	1.5	[53]
**2014**	643	−5.31	−3.50	3.39	0.71	27	0.65	[54]
**2015**	680	−5.3	−2.75	8.42	0.82	55	3.76	[55]
**2015**	652 *	−5.62	−3.52	10.48	0.9	56	5.29	[56]
**2015**	774	−5.23	−3.72	10.32	0.97	46.5	4.75	[57]
**2015**	614	−5.48	−3.44	10.20	0.90	55	5.05	[58]
**2015**	514 *	−5.33	−3.86	3.03	0.81	24	0.58	[59]
**2015**	655–792	−5.01	−3.73	13.39	0.73	37.3	3.6	[60]
**2015**	761	−5.34	−3.66	8.17	00.85	39	2.70	[61]
**2015**	696	−5.40	−3.81	7.64	0.73	38	2.12	[62]
**2016**	748	−5.19	−3.60	6.77	0.78	41	2.15	[63]
**2017**	627	−4.93	−3.28	13.79	0.768	66.5	7.2	[64]
**2017**	550	−5.11	−3.65	11.84	0.73	53.8	4.61	[65]
**2017**	765	−5.26	−3.91	13.9	0.64	65	5.8	[66]
**2017**	800	−5.23	−3.87	13.3	0.73	63	6.1	[42]
**2018**	668	−5.06	−3.60	12.98	0.7	62	5.61	[67]
**2018**	720 *	−5.39	−3.74	12.43 **	0.88	61	6.67	[68]
**2018**	752 *	−5.36	−3.79	14.32 **	0.95	67	8.98	[68]
**2018**	550–640	−5.37	−3.46	11.46 **	0.915	63	6.60	[39]
**2019**	717	−5.00	−3.42	5.17	0.672	40.8	1.62	[69]
**2019**	580	−5.28	−3.61	10.9	0.83	60	5.5	[70]
**2019**	725	−5.16	−3.43	7.72	1	31	2.79	[71]
**2019**	716	−5.47	−3.76	10.58	0.769	56.4	4.58	[72]
**2020**	538	−5.35	−3.08	2.27	0.67	27	0.37	[73]
**2020**	586 672 *	−5.16 −4.99	−3.17 −3.27	13.56 16.24	0.78 0.71	61 66	6.45 7.61	[29]
**2020**	586*	−5.91	−4.09	0.87	0.45	21	1.36	[74]

* In solution: ** After Solvent Vapor Annealing (SVA).

In Table 2, only devices with PCE above 5% are analyzed in detail. The table ranges from the least to the most efficient device. The parameters reported are the absorption window, the optical energy gap and the holes mobility of the molecule, and the geometry of the device with any thermal or solvent annealing treatments.

**Table 2:** Photophysical and photovoltaic characteristics of bulk heterojunction organic solar cells based on BODIPY small molecules with PCE above 5%.

Molecule [Ref.]	Optical Energy GAP [eV]	Treatment	PCE [%]	FF [%]	Hole Mobility [cm^2^/Vs]	Electron Mobility [cm^2^/Vs]	Abs. Band [nm]
**3** [58]	1.72	-	2.71	38	7.84 × 10^−6^	-	450–650
		TA	3.99	48	5.34 × 10^−5^	-	
		TA + SVA	5.05	55	8.45 × 10^−5^	-	
**4** [56]	1.84	NO	3.48	46	8.5 × 10^−6^	2.34 × 10^−4^	350–700
		Processed with pyridine 4% *v*/*v*	5.29	56	8.15 × 10^−5^	2.29 × 10^−4^	
**5b** [70]	1.59	Vacuum processed	5.5	60	9.2 × 10^−^^6^	-	350–1000
**6** [67]	1.74	-	2.74	34	-	-	300–850
		TA	4.72	57	2.09 × 10^−4^		
		TA +SVA	5.61	62	4.37 × 10^−4^		
**7** [66]	1.52	TA	5.8	65	0.8 × 10^−3^	-	400–800
**8** [42]	1.32	Vacuum processed	6.1	63	-	-	500–900
**9** [39]	1.79	-	2.54	39	5.34 × 10^−5^	2.35 × 10^−4^	400–700
		SVA	6.6	63	1.13 × 10^−4^	2.45 × 10^−4^	
**10** [63]	1.65	-	5.3	52	0.9 × 10^−4^	3.9 × 10^−4^	300–800
		TA	7.2	66.5	2.1 × 10^−4^	2.8 × 10^−4^	
**11a** [29]	1.78	-	3.21	46	-	-	300–600
		SVA	6.45	61	8.89 × 10^−5^	2.41 × 10^−4^	
**11b** [29]	1.58	-	3.76	48	/	/	300–700
		SVA	7.61	66	1.07 × 10^−4^	2.47 × 10^−4^	
**12a** [68]	1.52	-	3.32	41	3.95 × 10^−5^	2.31 × 10^−4^	400–800
		SVA	6.67	61	1.10 × 10^−4^	2.36 × 10^−4^	
**12b** [68]	1.44	-	4.73	45	4.85 × 10^−5^	2.38 × 10^−4^	400–800
		SVA	8.98	67	1.59 × 10^−4^	2.43 × 10^−4^	

**Figure 3:**
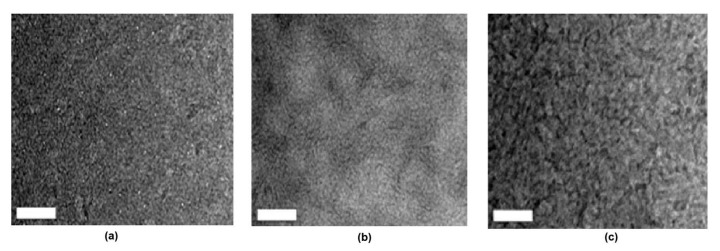
TEM images of the active layer **3**:PC71BM (**a**) as cast, (**b**) with thermal annealing (TA) and (**c**) thermal and solvent vapor annealing (TSVA). The scale bar is 200 nm. Reproduced from Ref. [58] with permission from the PCCP Owner Societies.

One of the first BODIPY small molecules based OSC with a PCE higher than 5% has been reported by Jadhav et al. [58] in 2015. The group proposes a new D–A type carbazole meso-substituted BODIPY small molecule (**3**, Scheme 1) as donor along with PC71BM as an electron acceptor in solution-processed BHJ solar cells. The molecule showed an absorption between 500 and 700 nm, with maxima at 568 and 620 nm and E_g_^opt^ of 1.75 eV. The best BHJ solar cell, with architecture ITO/PEDOT:PSS/active layer:PC71BM/Al (Figure 2b) shows a PCE of 2.71% which increases to 3.99% after thermal annealing (TA) and 5.05% after thermal and solvent vapor annealing (TSVA). The improvement in the efficiency, clearly induced by the TA and TSVA treatment, seems to be due to the increase in Jsc and the FF which originated from the enhanced absorption, better active layer nanoscale morphology (as shown in TEM images reported in Figure 3) and more balanced charge transport.

In the same year, Coutsolelos et al. [56] proposed a porphyrin-BODIPY-based small molecule as light-harvesting antenna system in BHJ solar cells (**4**, Scheme 1). The molecule consisted of a meso-aryl-substituted free base monocarboxy-porphyrin unit bridged by a central 1,3,5-triazine moiety to 4-aminophenyl-boron dipyrrin units through their arylamino groups. The absorption spectrum of the antenna system was the sum of the BODIPY and porphyrin absorption moiety, indicating that their connection through the triazine bridge does not cause significant electronic interactions between the two parts. In fact, the spectra showed in solution two main absorption maxima at 422 nm, attributed to the Soret band of porphyrin and 502 nm, attributed to the BODIPY unit, and other three less intense maxima (552 nm, 596 nm, and 652 nm) attributed to the Q-bands of the porphyrin unit.

**Figure 4:**
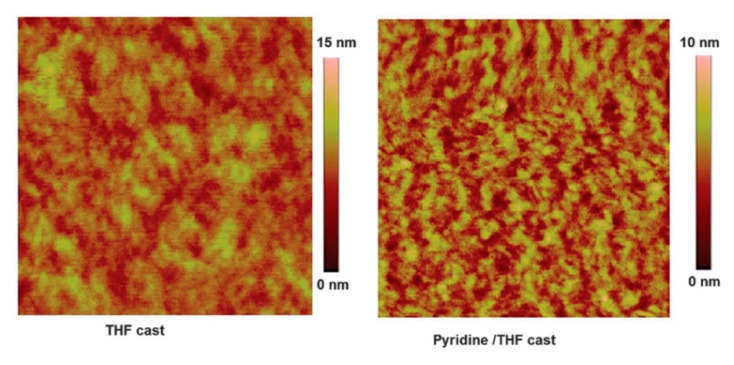
AFM images of **4**: PC71BM 1:1 blend thin films processed with and without pyridine additives. Image size: 3 mm. Reproduced from Ref. [56] with permission from The Royal Society of Chemistry.

This small molecule was tested in direct BHJ solar cells in blend with PC71BM, with architecture ITO/PEDOT:PSS/BODIPY:PC71BM/Al (Figure 2a). The optimized device, with donor/acceptor ratio 1:1 and processed with THF showed a PCE of 3.48% and an FF of 46%. To improve the performance of the devices, the active layer was processed with a solvent mixture of 4% *v*/*v* of pyridine in THF. The efficiency increased to 5.29%, with an FF of 56%, with an increase of an order of magnitude of the hole mobility. In this case, the enhancement can also be attributed to a better morphology and crystallinity of the active layer as evidenced in AFM images reported in Figure 4, leading to a better charge generation/separation/mobility/transport. 

The state of aggregation in the materials is another key parameter for understanding the performance of an OPV cell. Recently Leo et al. [70] have studied the effect of H- and J-aggregation [75] in NIR BODIPY-based small molecules BHJ solar cells. In the work, two BODIPYs with furan-fused pyrrole core structures, **5a** and **5b** in Scheme 1, possessing respectively H- and J-aggregation in neat film, have been studied. The two molecules showed similar absorption spectra in solution, while in solid-state **5a** preferred a cofacial H-aggregation and **5b** adopted a J-aggregation, as a result, the solid-state spectra are significantly different. Both BODIPYs presented broadened bands with absorption from 450 to 900 nm. Compared to the solution, the absorption maximum of **5a** is hypsochromically shifted at 580 nm, with a shoulder peak at 417 nm, while **5b** showed bathochromically shifted absorption maximum at 777 nm with a shoulder peak at 630 nm. The two molecules have been tested in vacuum-deposited BHJ solar cells, with architecture ITO/MH250:W_2_(hpp)_4_ (5 nm, 7 wt%)/C70 (15 nm)/BODIPY: C60 (1:2 *v*/*v*, 30 nm, 95 °C)/BPAPF (5 nm)/BPAPF:NDP9 (40 nm, 10 wt%)/NDP9 (1 nm)/Al (100 nm); where W_2_(hpp)_4_ (tetrakis(1,3,4,6,7,8-hexahydro-2*H*-pyrimido[1,2-a]pyrimidinato)ditungsten (II)) and MH250 (*N*,*N*-bis(fluoren-2-yl)-naphthalenetetracarboxylic diimide(bis-Hfl-NTCDI)) are used as electron transporting layer (ETL), and NDP9 (Novaled AG) doped BPAPF (9,9-bis[4-(*N*,*N*-bis-biphenyl-4-yl-amino)phenyl]-9*H*-fluorene) served as hole transporting layer (HTL) (Figure 2e). The intrinsic layers adjacent to the n-/p-doped layers worked as exciton reflection and hole/electron blocking layer. The C70 intrinsic layer also contributed to the photon absorption and photocurrent generation. The two molecules showed good performances, in particular, **5b** showed an efficiency of 5.5% with an FF of 60%, while **5a** showed a lower efficiency of 4.2% and an FF of 55%. The better performance of 5b may be attributed to bathochromic shifted absorption, a small driving force of exciton dissociation, and lower nonradiative voltage loss, compared to 5a.

**Figure 5:**
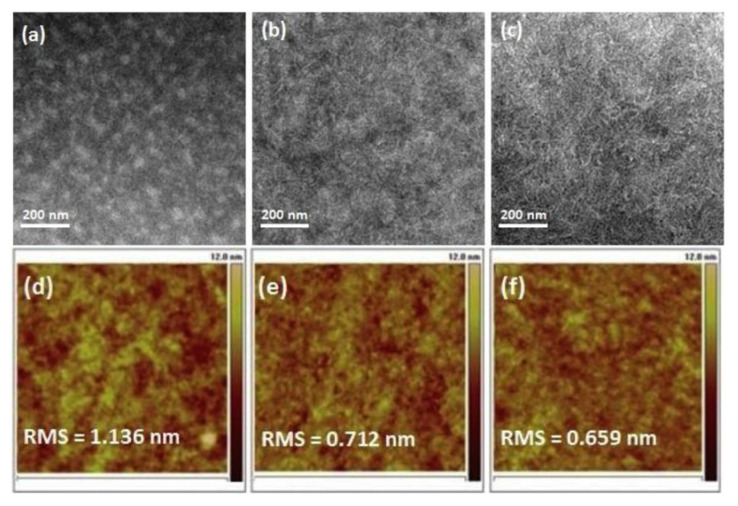
TEM images and AFM height images of an active layer based on **6**:PC71BM: (**a**,**d**) as cast; (**b**,**e**) with TA; (**c**,**f**) with TA and solvent vapor annealing (SVA) treatments**.** Reproduced from Ref. [67] with permission from 2018 Elsevier B.V.

Fang et al [67] in 2018 reported a novel donor–acceptor–donor (D–A–D) type small molecules consisting of a BODIPY linked through alkynyl with two benzo[1,2-b:4,5-b’]dithiophene (BDT) terminal donors for solution-processed BHJ solar cell (**6**, Scheme 1). The molecule showed an absorption spectrum, in thin-film, from 300 to 800 nm with a maximum at 668 nm and an optical energy gap of 1.46 eV. The small molecule has been tested as an electron donor in solution-processed BHJ with architecture ITO/PEDOT:PSS/active layer/Ca/Al. The optimized device with thermal annealing treatment, showed a PCE of 4.72%, with an FF of 57%. In this case, the efficiency can also be increased up to 5.61% with an FF of 62% when used both thermal annealing and solvent vapor annealing treatment. The improved efficiency can be attributed to a better morphology and reduced roughness from 1.136 to 0.659 nm as evidenced by TEM and AFM reported in Figure 5.

To increase the molecular packing Bulut et al. [66], inspired by Leclerc and his research group, proposed a BODIPY linked to two triazatruxene (TAT) derivatives as planar end groups (**7**, Scheme 1). This kind of architecture (TAT–dye–TAT) would lead to strengthened molecular stacking behavior, while the optical properties were still essentially determined by the central dye. Moreover, the addition of a TAT platform was able to favor the out-of-plane hole mobility. This small molecule was one of the rare examples of inverted architecture, in fact, it has been tested with the following geometry ITO/PEIE(5 nm)/BODIPY:PC71(61)BM/MoO_3_(7 nm)/Ag(120 nm) (Figure 2c) reaching an efficiency of 5.8% and FF of 65%. A hole mobility of 0.8 × 10^−3^ cm^2^ V^−1^ s^−1^ was measured using hole-only space-charge limited-current (SCLC) devices.

Another successful strategy to increase the efficiency of solar cells has been to increase the absorption window by developing dyes capable of absorbing in the IR region. A BODIPY with intense and long-wavelength absorption can be achieved by an extension of the π-system and an electron-withdrawing group on the meso-position. Li et al. [42] have developed a BODIPY dye able to combine the absorption in a wide spectral window with an excellent packing reaching an efficiency of 6.1%, with an FF of 63%. The synthesized material was a NIR furan fused BODIPY with a CF_3_ group in meso-position (**8**, Scheme 1). The small molecule showed an absorption spanning from 500 nm to 900 nm and absorption maxima at 712 nm and 800 nm in the solid-state. The choice of methoxyl group on the peripheral phenyl rings influenced the packing behavior and a brickwork-type arrangement in the neat film is observed, comprising an alternating arrangement of a unit consisting of two antiparallel molecules. Consequently, **8** had a J-aggregation character, moreover, charge transport in OSCs benefits from aggregation, leading to higher overall PCE through improved Jsc and FF, even though Voc (open circuit Voltage) may decrease slightly. The new BODIPY derivatives have been tested as an electron donor in vacuum processed n-i-p BHJ solar cells, with the architecture reported in Figure 2e ITO/MH250:W_2_(hpp)_4_ 7 wt% (5 nm)/C70 (15 nm)/**8**:C60 (40 nm)/BPAPF (5 nm)/BPAPF:NDP9 10 wt% (40 nm)/NDP9 (1 nm)/Al (100 nm). W_2_(hpp)_4_ and NDP9 were n- and p-dopants and MH250/BPAPF was an electron/hole transporting material. The maximum PCE of 6.1% has been obtained after thermal annealing at 100 °C. The high EQE and Jsc (Short circuit current density) of the device indicate that the photogenerated excitons are efficiently separated into free charge carriers. AFM analysis confirmed that **8** and C60 were well intermixed, providing enough D/A interfaces for exciton dissociation. The molecule has been tested also in tandem with solar cells, in combination with the green absorber DCV5T-Me. In this case, the tandem solar cell has achieved an efficiency of 9.9% and FF of 59%.

**Figure 6:**
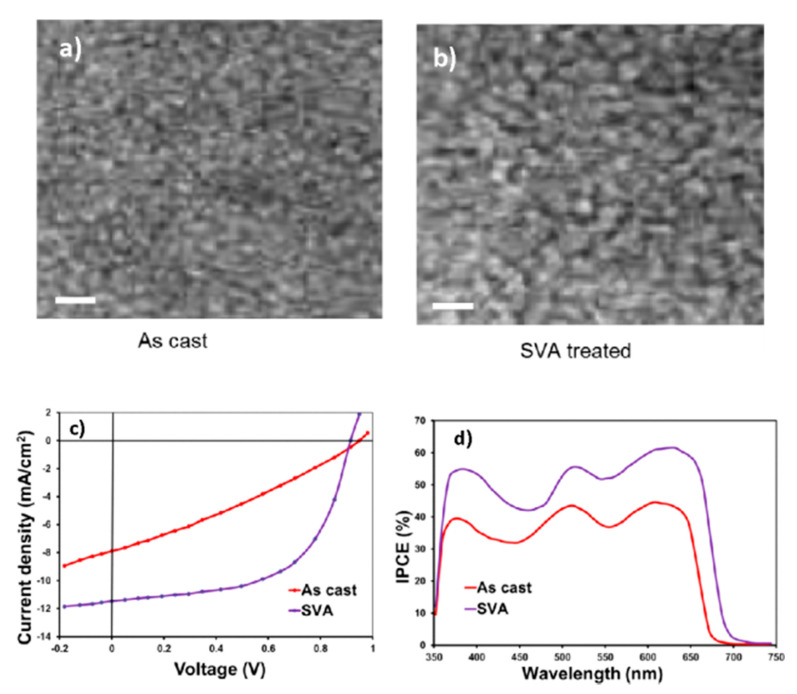
TEM image of as-cast 88 (**a**) and SVA-treated (**b**) **9**:PC71BM thin films (Scale bar is 100 nm). (**c**) Current−voltage characteristics under illumination and (**d**) IPCE spectra of the OSCs base on as-cast and SVA-treated **9**:PC71BM active layers. Reproduced from Ref. [39] with permission from 2018 American Chemical Society.

Sankar et al. [39] have proposed a corrole-BODIPY derivative as an electron donor in solution-processed BHJ. The molecule consisted of a central Ga(III) corrole with two peripheral BODIPY units (**9**, Scheme 1). The two strong absorption bands of the molecule in thin films centered at 432 and 516 nm are attributed to the Soret bands of corrole and BODIPY and the broadband between 550–640 nm, is attributed to Q-bands typical of corrole-based systems [76]. The molecule has been tested as an electron donor in BHJ with structure ITO/PEDOT:PSS/BODIPY:PC71BM/PFN/Al, where poly[9,9-bis(3′-(*N*,*N*-dimethylamino)propyl)-2,7-fluorene] (PFN) is used as the cathode interlayer (Figure 2b). The optimized device, with active layer as cast, had an efficiency of 2.54% and FF of 39%, while the optimized device a, with solvent vapor annealing treatment, showed an efficiency of 6.60 and FF of 63%. As shown in Figure 6, better phase separation was found for the SVA-treated active layer compared to the as-cast active layer, forming more bicontinuous interpenetrating networks, which was beneficial for charge-transport efficiency, leading to improved J_sc_ and FF.

**Figure 7:**
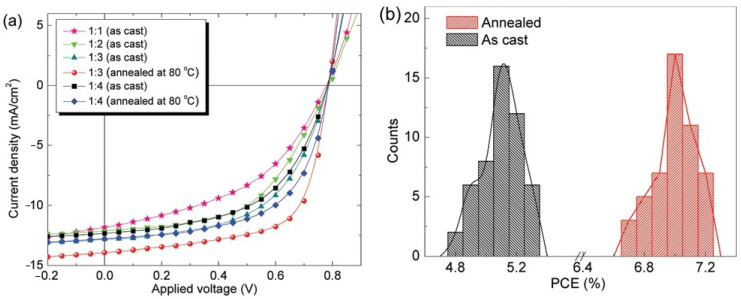
(**a**) The light J–V characteristics of bulk hetero junction (BHJ) organic solar cells fabricated with “as cast” and “annealed” **10**:PC71BM blend photoactive layers and (**b**) the efficiency histogram of both categories of devices. Reproduced from Ref. [63] with permission from The Royal Society of Chemistry.

In 2017, R. Srinivasa Rao et al. [63] reached one of the highest efficiencies in BODIPY-based solar cells. The groups synthesized a BODIPY molecule decorated with dithiafulvalene wings with a broad absorption profile from 350 to 780 nm and extending up to the near IR region (**10**, Scheme 1). The solution process BHJ device, with architecture ITO/PEDOT:PSS/active layer/Ca/Al (Figure 2b) was prepared with the active layer obtained as cast and reached, after thermal annealing 7.2% of PCE with a FF of 66.5% stressing once again the importance of controlling the nano-scale aggregation. The authors have reached this excellent result for a series of positive factors such as the large absorption window, suitable HOMO and LUMO energy levels, excellent affinity with the acceptor, and an optimal nanomorphology (Figure 7).

Recently, Yang et al. [29] have designed and synthesized two carbazole–BODIPY-based dyes by Knoevenagel reaction, the first bearing one carbazole unit in position 3, the second bearing two carbazole unit, in position 3 and 5 respectively (**11a** and **11b**, Scheme 1). The introduction of carbazole donating unit in D–A organic semiconductors, lowering the HOMO energy level, resulted in a high open-circuit voltage in OSCs in addition, remarkably red-shifted absorption spectra and better hole transport are reported. The molecules showed classic absorption spectra for BODIPY, with the maxima at 586 nm and 672 nm in DCM solution and broadening and red shift in thin films due to aggregation. The molecules have been tested in solution-processed BHJ solar cells with architecture ITO/PEDOT:PSS/active layer:PC71BM/PFN/Al (Figure 2b), using a conjugated polar polymer for charge regulation at the active layer/electrode interface as previously reported by Sankar and co-worker. In this way the BHJ solar cell reached after solvent vapor annealing, the efficiency of 6.45% and 7.61% and FF of 61% and 66% respectively for the molecules with one carbazole and two carbazole units. Once again, an enhanced crystalline nature and reduced π–π stacking distance for the **11b**:PC71BM active layer may induce better nanoscale phase separation and may be beneficial for charge transport and collection. These observations may be the reason for the higher FF and greater PCE of the OSCs based on the latter.

**Figure 8:**
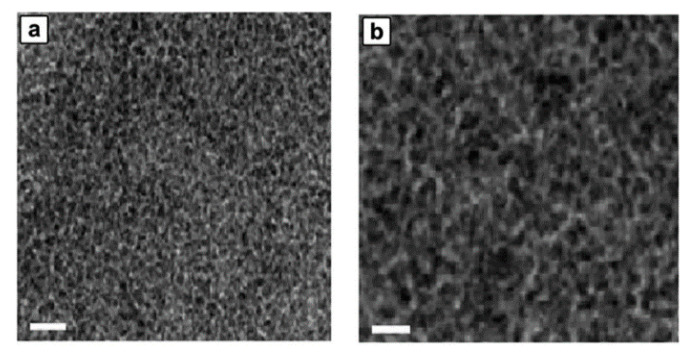
Transmission electron microscopy (TEM) images of optimized (**a**) **12a**:PC71BM and (**b**) **12b**:PC71BM thin films. The scale bar is 100 nm. Reproduced from Ref. [68] with permission from The Royal Society of Chemistry.

**Scheme 2:**
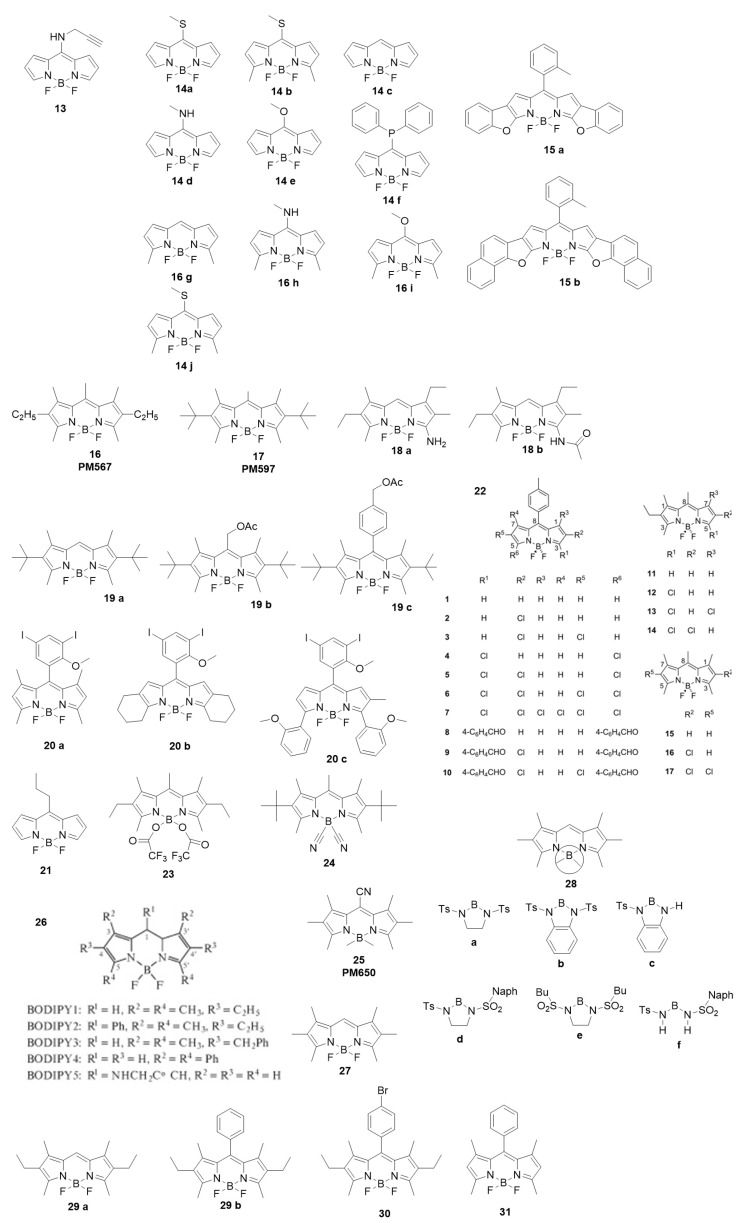
Chemical structure of BODIPY-based molecules as active materials in solid-state photonic devices. Molecule **22** reproduced from Ref. [77] with permission from 2012 WILEY-VCH Verlag GmbH & Co. KGaA, Weinheim, Germany. Molecule **26** reproduced from Ref. [78] with permission from 2014 Turpion Ltd.

The highest efficiency for BODIPY small molecule-based OSC was obtained by Bucher and co-worker [68] in 2018. The group designed and synthesized two small molecules comprised of a central BODIPY core, surrounded with two diketopyrrolopyrrole (DPP) and two porphyrin side units, differing only from each other in the aromatic bridge between BODIPY and porphyrins: in one case phenylene (**12a**, Scheme 2) was used, in the other case thienyl (**12b**, Scheme 1). In both cases, complementary absorption properties of the three units allowed a wide solar photons harvest and, as matter of fact, the small molecules showed a panchromatic absorption spectrum from 400 nm to 800 nm in the thin film and an optical E_g_ of 1.52 and 1.44 eV respectively for **12a** and **12b**. The OSC, with architecture ITO/PEDOT:PSS/BODIPY:PC71BM/PFN/Al (Figure 2b), where the BODIPY is the **12a** molecule, after solvent vapor annealing, presents a PCE of 6.67% and FF of 61%, while with the molecule **12b** has a record efficiency, after SVA, of 8.98% and a FF of 67%. The different performances in OSC might be attributed to the different morphology in the blend, as reported in Figure 8 where the presence of nanofibril and phase separation has increased the charge transport in the layer and subsequently the PCE of the device.

From the results reported in Table 2, it is evident that in a solar cell the parameters to be taken into consideration are manifold and all closely connected to each other. Among them, two proved to be particularly important for the development of this class of materials, the absorption spectral window that must clearly be as wide as possible and the active layer morphology. The latter strongly affects the efficiency of exciton dissociation, the charges mobility, and their recombination. For this reason, it is worth to stress the thermal treatments importance and the solvent annealing. We have shown that for the same material, once TA and SVA have been made, the solar cell efficiency can often be doubled.

## 3. Photonic Applications

Photonics play an important role in driving innovation in an increasing number of fields. The application of photonics spreads across several sectors: from optical data communications to imaging, lighting, and displays; from the manufacturing sector to life sciences, health care, security, and safety. In this context, organic semiconductors are promising candidates to fulfill the capacity of photonics and deliver on their promises. Among all the organic molecules BODIPY derivatives seem to have all the requisites to be effectively used as active materials in photonics. As previously mentioned BODIPY is characterized by a high photoluminescence quantum yield and, it can be easily functionalized on several different sites allowing a fine modulation of its photophysical properties.

BODIPY derivatives have been found to be suitable materials for the development of tunable organic lasers. Diluted solutions of those organic dyes have been exploited as lasing media with excellent results, comparable and even better than most common and largely used dyes such as coumarin, rhodamine, and polymethine [9,79]. Despite these results, there have been only a few studies centered on the solid-state; this was probably related to two main reasons: in the solid-state the quantum yield is usually lower than in solutions and BODIPY complexes seem to be particularly affected by the composition and structure of the polymer matrices in which they generally need to be dispersed to maintain the most of their properties.

For this reason, in view of a possible optimization of the use of this material in this review, we focus on their performances as solid-state depending on different groups or atoms substitutions (the chemical structures of the BODIPY-based molecules have been reported in Scheme 2).

### 3.1. Fluorescence Emission Optimization in BODIPY Derivatives

In solid-state BODIPY derivatives, the fluorescence is normally strongly quenched mainly due to intermolecular interactions. This is strongly detrimental for photonics applications. The suppression of intermolecular interactions proved to be a useful and valid strategy for decreasing quenching and increasing quantum yield [10]. In the next section, the main researches performed to control interactions between BODIPY molecules and to tune materials emission properties have been reported.

The first method to reduce intermolecular close packing (π-π interactions) is to introduce a bulky group playing as a “spacer” [80,81]. Following this strategy, BODIPY-based emissive compounds have been synthesized by substitution with bulky spacers such as bulky tert-butyl substituents on the meso-phenyl groups [82], bulky arylsily groups [83], or adamantyl group in the 3 and 5 positions of the BODIPY core [84].

**Figure 9:**
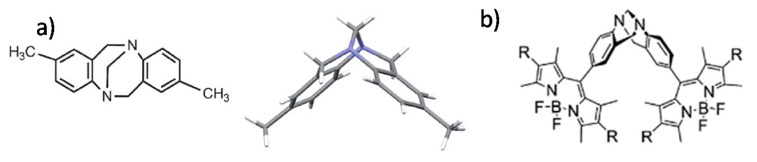
Structure of Tröger’s base (**a**) and structure of the new BODIPY (**b**). Reproduced from Ref. [85] with permission from The Royal Society of Chemistry.

A second, different approach is to attach the planar BODIPY core to a twisted or nonplanar scaffold. As an example, a Λ-shaped Tröger’s base (Figure 9) skeleton was introduced in the BODIPY core [85]. The new-synthesized dye displayed an intense fluorescence, indicating that quenching caused by aggregation, was suppressed.

A further factor that directly influences the intensity of the fluorescence is the intersystem crossing process between the singlet and the triplet states. This process can be eliminated by making the dyes skeleton more rigid [86] leading, however, to a tighter packing and so to detrimental interchain interactions. A delicate balance between these 2 effects is required. Swamy et al. [87] showed that the molecular free rotation and aggregation-induced fluorescence quenching of BODIPYs can be successfully suppressed by lowering the flexibility of the molecules with a series of appropriate substitutions in the dye.

**Figure 10:**
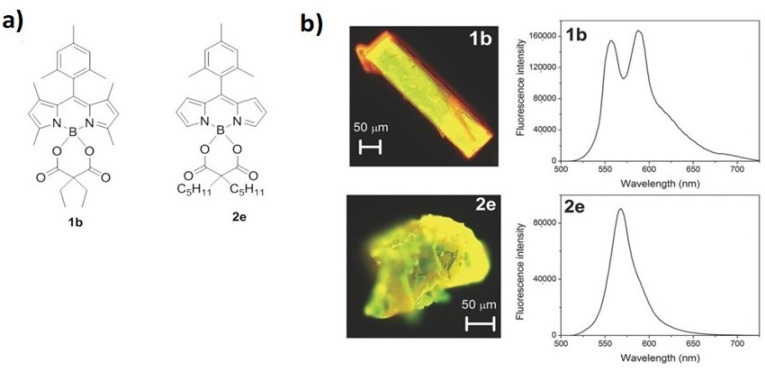
(**a**) Structure of compounds 1b and 2e. (**b**) Fluorescence images and spectra of solid crystals of representative compounds 1b and 2e were recorded under excitation at 450–490 nm with a fluorescence microscope. Reproduced from Ref. [88] with permission from 2016 WILEY-VCH Verlag GmbH & Co. KGaA, Weinheim, Germany.

Aggregation is not always detrimental to the fluorescence quantum yield. In particular, J-aggregation can have a fundamental role and be responsible for red-shifted emission. Taking advantage of this feature, Manzano et al. [88] designed new O-BODIPY derivatives with spontaneous formation of stable J-aggregates and characterized by a conformational rigidity due to the presence of the *B*-spiranic structure and the disposition of the *B-*diacyloxyl substituent and the meso-aryl group. So, by controlling the J-aggregation process, it was possible to tune dyes properties, allowing the excitation of efficient and tunable laser emission of both the monomeric and J-aggregated forms. Moreover, by playing with the conformational rigidity of the system, the fluorescence quantum yield was improved, by reducing quenching due to H-aggregates. The authors observed highly efficient and stable red-shifted laser emission from J-aggregates in pure organic solvents and solid-state crystals. Figure 10 shows the emission properties of two representative dyes: 1b with J-aggregation capability and 2e with monomeric units. We observe that, after UV light excitation, both crystals display fluorescence emission, but efficiencies and spectral profiles are very different. This is related to the different molecular arrangement: 2e exhibits a single emission corresponding to the monomer, while in 1b we have a second long-wavelength emission, assigned to the corresponding J-aggregated form.

The impact of aggregation on luminescence was further investigated by Bozdemir et al. [89] studying the emission variation related to different distributions of crystalline and amorphous regions while the range of states produced by system self-assembly (from extended aggregates to other different structures) and influenced by side-group structure was investigated by Musser et al. [90]. In another work, the effect of spiro-structures in J-aggregate formation was studied. It was found that spiro-bicyclic structure has an efficient role in preventing face-to-face π-stacking interactions, which is frequently observed in solid-state BODIPYs, and as a consequence, it can be used as a strategy to enhance the emission intensity [91].

### 3.2. BODIPY Derivatives as Lasing Dyes

Fluorescent BODIPY derivatives emitted from the green spectral region, above 500 nm, to the near infra-red (NIR) range between 650 and 900 nm; furthermore, functionalization on the 8 meso-position shifted the emission wavelength in the blue spectral region. Therefore, this rather young class of dye materials presents a broad and tunable emission spanning from the blue to the NIR. Here below we describe three research studies where the BODIPY derivative was in solution because we consider them as important landmarks to understand the following studies in solid-state.

In 2010, Gómez-Durán and co-workers [92] synthesized a new dye by incorporating a propargylamine group at the meso-position of the BODIPY core (**13**, Scheme 2). This incorporation leads to a hypsochromic shift of the absorption and fluorescence bands to the blue part of the visible region. The new BODIPY structure exhibits a fluorescence quantum yield up to 0.94 and a laser emission tunable between 455 and 515 nm depending on the solvent. Even if the new dye photostability is still quite-low, this work represents an important benchmark for the capability of tuning BODIPY laser emission.

**Figure 11:**
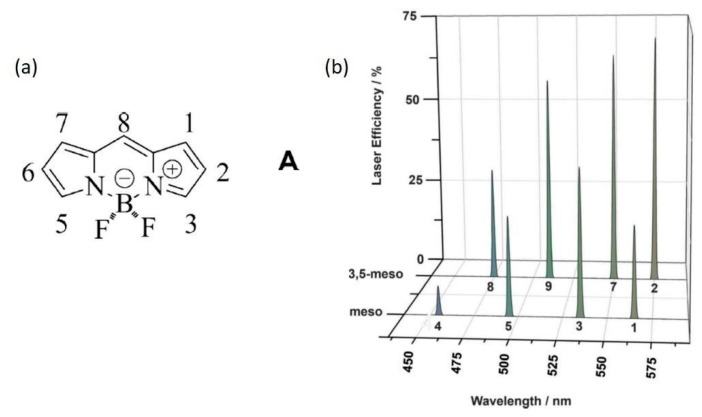
(**a**) BODIPY core structure. (**b**) The efficiency of the laser emission of the different dyes meso-substituted and 3,5-meso-substituted in ethyl acetate solution. The numbers under the peaks identify the corresponding dye. Reproduced from Ref. [93] with permission from 2013 WILEY-VCH Verlag GmbH & Co. KGaA, Weinheim, Germany.

In 2013, Esnal et al. [93] published a work where they presented BODIPY derivatives with unprecedented laser efficiencies and good photostabilities. In their study, they developed a series of meso-substituted BODIPY dyes. Starting from the core structure A (Figure 11a), they increased efficiency by adding methyl groups at positions 3 and 5 and they changed the laser emission region by changing the heteroatom at position 8 (N, S, O, P, and H) (**14**, Scheme 2). As shown in Figure 11b, the laser efficiency and the emission wavelength depend on the heteroatom incorporated at position 8. Moreover, the laser emission efficiency is greatly improved by incorporation of methyl groups at positions 3 and 5 and in some dyes can reach also a value higher than 70%.

**Figure 12:**
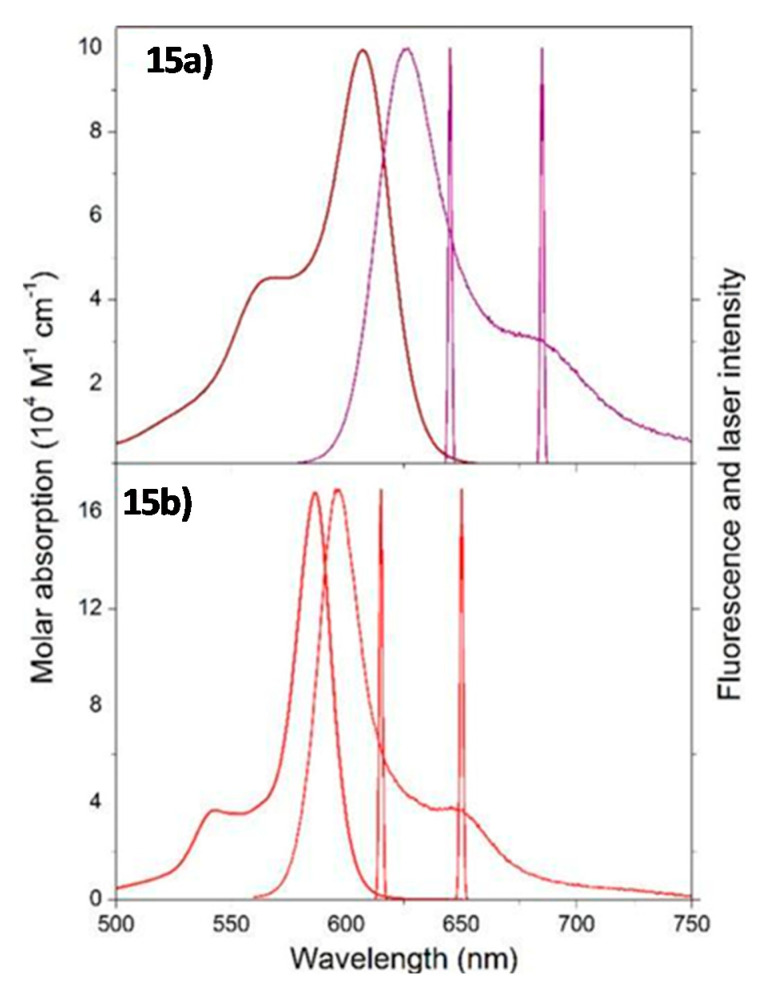
Absorption, normalized fluorescence, and laser (dual emission depending on the dye concentration) spectra of dye **15a** and its analog **15b**, with additional fused phenyl in ethyl acetate. Reproduced from Ref. [94] with permission from 2019 American Chemical Society.

The last important step was made by the work performed by Belmonte-Vázquez et al. [94], who developed a new family of benzofuran-fused BODIPY dyes (**15**, Scheme 2) reaching very high fluorescence and laser efficiencies (almost 100% and >40%, respectively); moreover, they obtained emission with a deep shift to the red edge related to the promotion of excited state aggregates at high concentrations (Figure 12). The efficiency and photostability of these new BODIPYs made it possible to overcome the limits of most of the commercially available dyes (such as cyanines or oxazines) in the same spectral region.

The transition from measuring lasing emission in solutions to have the same in solid-state was not straightforward. In principle, many are the advantages of using polymeric materials as a solid “environment” for lasing dyes: they are optically homogeneous, characterized by good chemical compatibility with organic dyes, and, as it is for solvents, they can be used to control medium polarity and viscoelasticity. On the other hand, these materials are limited by photodegradation processes. To optimize lasing properties and photostability, many new materials based on organic compounds have been synthesized and characterized by the photophysical point of view. In general, BODIPY-based dyes used for solid-state lasing must be dispersed in matrices. This allows for the avoidance of quenching linked to oxidation and in some cases to protect them or slow down the oxidation phenomena.

A key factor in the choice of matrices is also the presence of polar groups (their polarity) which has been shown to have a strong impact [95] on both the stability and efficiency of the films. Studies have been carried out to analyze the dependence of laser properties on several experimental parameters as a dye, pumping repetition rate, the composition of the matrix hosting the dye. Lasing performances have been evaluated by measuring the energy conversion efficiency, given by the ratio between the energy of the laser output and the energy of the pump laser impinging on the sample surface, the laser emission spectrum, and the photostability, which gives us the evolution of the laser output as a function of the number of pump pulses in the same position of the sample at a selected repetition rate.

**Figure 13:**
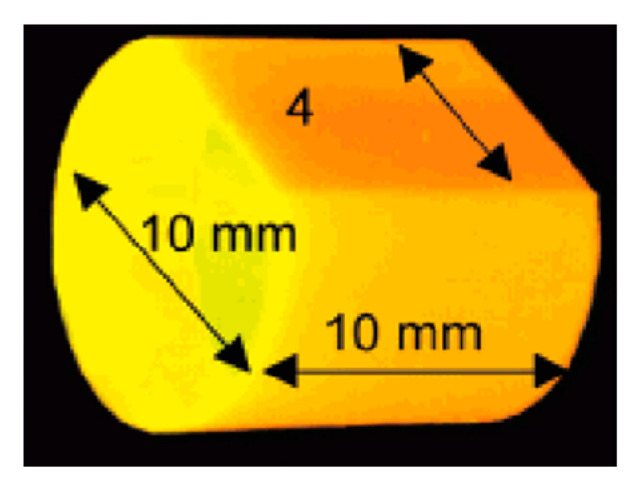
Polymeric solid laser sample. Reproduced from Ref. [96] with permission from 2007 American Chemical Society.

In the beginning, measurements for laser characterization were performed on a “rod” sample of the dye matrix. As described by Costela et al. [96], the solid laser samples were constituted by cylindrical rods of around 10 mm diameter and 5–10 mm length (shown in Figure 13). In their work, Costela et al. studied the commercial BODIPY derivatives pyrromethene 567 (PM567, **16** in Scheme 2) dissolved in copolymers of MMA with different monomer compositions. After transversely pumping at 534 nm at 5.5 mJ, the lasing efficiency was measured up to 30% with a good photostability.

In the following decade, many studies were performed by changing the BODIPY derivative used as a laser dye (summarized in Table 3).

In 2007, the work of Garcίa et al. [95] revealed that laser efficiency of commercial BODIPY derivatives PM567 and PM597 (**16** and **17**, Scheme 2) in organic polymeric matrixes was deeply increased by the incorporation of fluorine atoms in the structure of the organic matrixes increasing both photostability and efficiency providing useful information on the potential of the choice of different materials as photostable laser media. They reached up to a 36% and 42% laser efficiency for PM567 and PM597, respectively and they obtained a high laser photostability; thus, paving the way for the development of industrial oriented laser applications of organic solid-state materials for telecommunication, instrumentation, and display technologies.

In the same year, Amat-Guerri’s team [97] established an easy synthetic method for the synthesis of asymmetric 3-amino- and 3-acetamido-BODIPY dyes from 2,3,4-trialkyl-substituted pyrroles (**18**, Scheme 2). They obtained materials with good laser emission properties mostly in ethanol solutions but also in solid-state, with an efficiency of up to 48% and 25%, respectively.

A few years later, Costela et al. [98], worked on a simple protocol to synthesized BODIPY with tunable absorption and emission bands in the visible and NIR spectral range. Starting from the commercial PM597 dye, the novel dyes were built by changing the group present at the meso-position: the 8-methyl group was substituted with 8-hydrogen (**19a**, Scheme 2), 8-acetoxymethyl **19b**, Scheme 2), or 8-*p*-acetoxymethylphenyl (**19c**, Scheme 2) groups. The differences induced by those changes were studied; a tunable laser action was observed and the novel analogs showed a high (up to 48%) and photostable laser emission: these results have been so good to consider the new dyes as benchmarks in the extended spectral region (from the green-yellow to red range).

**Figure 14:**
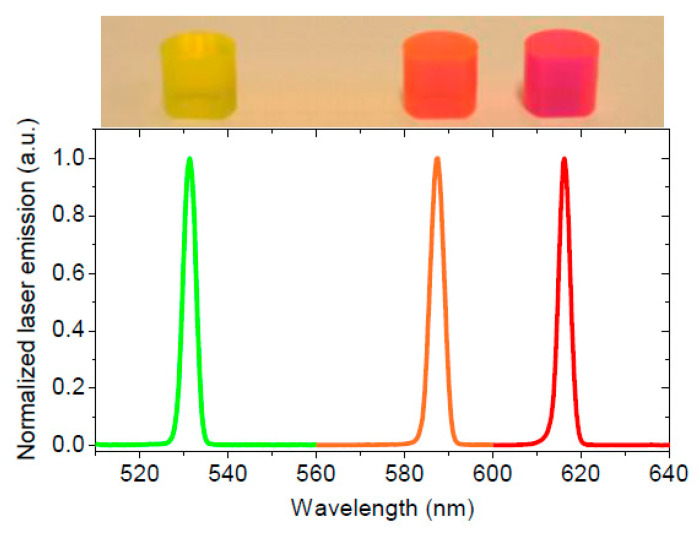
New photosensitized materials based on the new dyes incorporated into PMMA: lasing emission spectra of dyes **20a** (green), **20b** (orange), and **20c** (red). Reproduced from Ref. [99] with permission from 2011 Optical Society of America.

Tunability of laser emission in solid-state was extended even more by Pérez-Ojeda et al., who in 2011 published a work with the synthesis and study of diiodinated BODYPY derivatives [99] (**20**, Scheme 2) both in solution and incorporated into polymeric matrices. They modulated the position of the emission band by choosing the type of substituent attached to the BODIPY core. They found high laser efficiency and photostability of the dyes exhibiting laser emission from 530 nm to 625 nm (see Figure 14) with notable efficiencies, up to 55% in solutions and 45% in PMMA. Moreover, the laser action of these novel BODIPY derivatives were improved in comparison with the lasing properties of commercially available BODIPY dyes (such as PM567, PM597, and PM650), which when incorporated into PMMA, showed lasing efficiencies of 28%, 40%, and 13%, respectively.

The same group combining the results obtained on fluorinated matrices [95] and on the development of BODIPY dies reported efficient and stable laser emission from BODIPY dye incorporated into polymeric matrixes after high UV pumping excitations [100]. In their study, they synthesized 8-propyl-4,4-difluoro-4-bora-3a,4a-diaza-s-indacene (**21**, Scheme 2), which was then incorporated into PMM), which mimics ethyl acetate solvent, and copolymers with various volumetric proportions of monomers MMA and 2-trifluoromethyl methacrylate (TFMA). Under pumping at 355 nm, lasing efficiencies up to 29% were measured (similar to those obtained in solutions). Moreover, **21** showed photostability higher than what was usually achieved with commercial dyes emitting in the same spectral region, for example, Coumarin 540A. The results pointed out the potential of these class of novel dyes as they are characterized by a relatively easy synthetic buildup with a large variety of possible substituents that can be used to tune the range of solid-state dye lasers to the blue region of the visible range.

Regarding the enhancement of laser properties of BODIPY derivatives with respect to their commercially available analogs, a second work was published in 2012 by Durán-Sampedro et al. [77]. In the article, the novel chlorinated BODIPYs (**22**, Scheme 2) exhibited enhanced laser action in solution and the solid phase. Pumped at 532 nm, the chlorinated BODIPY derivatives showed high efficiency (up to 30%) and photostable laser emission centered between 565 and 615 nm. Fluorescence QY was increased by introducing electron acceptor chlorine substituents in the 3- and 5-positions. As a consequence, lasing properties are enhanced both in a liquid solution and in the solid phase. This is related to the improvement in the photophysical properties of the molecules and in particular for their dipole moments. In fact, the dipole orientations are modified to match pump laser polarization, leading to significant enhancement of system efficiencies.

Up to now, we have reported studies performed in the conventional two-mirror lasers with a thick (bulky) sample. Nevertheless, from 2000, there has been significant work to develop organic thin film (TF) lasers based on dye-doped polymers. It was clear that TF lasers have great potential applications as coherent light sources to be integrated into optoelectronic devices, spectroscopy, and sensors.

In 2013, Durán-Sampedro et al. [78], implemented newly synthesized BODIPY in distributed feedback (DFB) lasers, the most common resonator type for organic thin-film lasers. They developed O -BODIPYs from commercial dyes by replacing fluorines with electron acceptor carboxylate groups (**23**, Scheme 2). They found that in solid-state samples the O-BODIPY derivatives with electron acceptor carboxylate groups with PMMA as matrices, lasing efficiencies were higher than what measured in the corresponding commercial dyes, with the best result up to 56%. A similar outcome was reported also in DFB lasers: in O-BODIPY dyes, laser thresholds were lower than what was found in the parent dyes, and moreover, lasing intensities were up to one order of magnitude higher.

**Figure 15:**
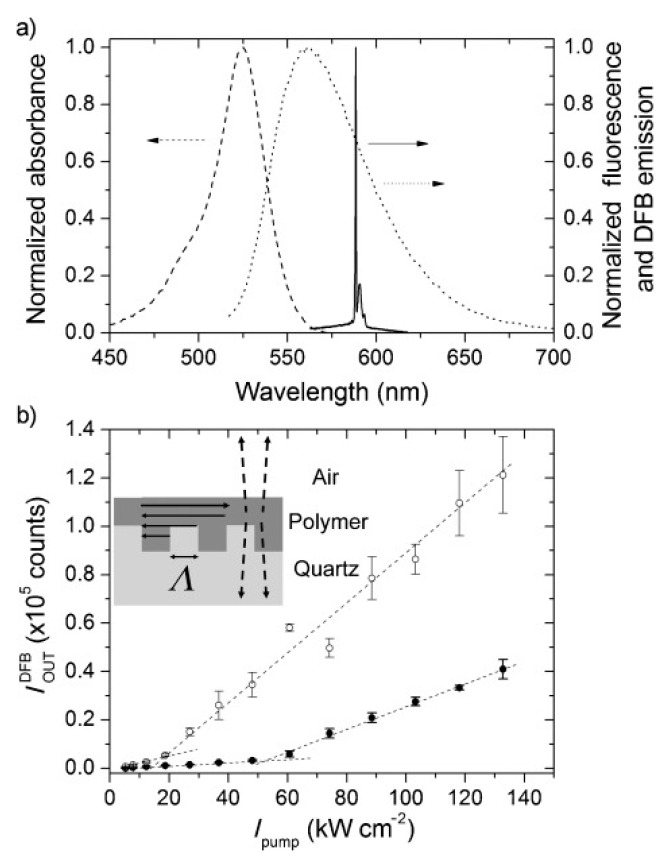
(**a**) Absorption (dashed line), fluorescence (λexc = 500 nm, dotted line), and laser spectra (solid line) of derivative **24** (23 mm) doped in PMMA. (**b**) DFB output intensity as a function of pump intensity from thin films of commercial PM597 (**17**, Scheme 2) 19 mm (filled circles) and derivative **24** (23 mm; hollow circles) doped in PMMA. Inset: sketch of DFB laser. The arrows represent in-plane feedback owing to second-order diffraction (solid arrows) and outcoupled laser emission owing to first-order diffraction (dashed arrow). Reproduced from Ref. [101] with permission from 2014 WILEY-VCH Verlag GmbH & Co. KGaA, Weinheim, Germany.

One year after, the same group synthesized a new library of BODIPY derivatives [101] with different Boron-substituent groups (alkynyl, cyano, vinyl, aryl, and alkyl) (**24**, Scheme 2). They prepared both bulk solid-state samples (Table 3) and TFs (Table 4) of the new dyes incorporated in organic matrices. Even in this case, the new classes of BODIPY showed better performances than the parent dyes. In bulk solid-state, good lasing efficiencies (up to 53%) were gained; while in TFs, the authors found lower laser thresholds and greater lasing intensities, up to three orders of magnitude higher as shown in Figure 15.

In 2014, an interesting work was published on the relationship between small modifications in the material used as a matrix in DFB laser and laser performances of different dyes [102]. The authors slightly modified the commercially available PAZO-Na azobenzene and they used the obtained new materials (carboxylic acid and different salts) as matrices for several common laser dyes: rhodamine, DCM, kiton red, pyrromethene, pyridine, LDS, and exalite. They found that even small modifications in PAZO-Na deeply affect laser stability and efficiency, which are strongly improved due to changes in polarity, refractive index, and morphology of the polymer matrix used. In particular, for PAZO-H, laser generation with the commercial BODIPY dye PM650 (**25** Scheme 2), has been detected, which was attributed to the lower polarity of the matrix while with other PAZO-Na derivatives no lasing was measured with the same dyes. This indicates that in BODIPY dyes, lasing can be easily optimized by changing matrix parameters such as polarity.

In 2014, also Kuznetsova et al. [103] exploited how modifications of the matrix impacted dye lasing efficiency and other parameters. In their research, they incorporated different BODIPY complexes (**26**, Scheme 2) into bulk PMMA matrix and matrix modified with the addition of polyhedral silsesquioxane particles. The interesting result was that modification of the matrices affected only slightly the spectral features but instead, it significantly improved the lasing efficiency and threshold. As an example, it has been reported that in a modified matrix a simple BODIPY derivative (3,3′,5,5′-tetramethyl-4,4′-diethyl-2,2-dipyrromethene difluoroborate, BODIPY1) shows a maximum efficiency up to 90% while 70% in the unmodified matrix.

Further encouraging results obtained by implementing the laser media have been reported in 2016 by Kuznetsova et al. [104] by incorporating alkyl BODIPY derivatives (**27**, Scheme 2) into three component silicate demonstrated the possibility of developing solid-state laser media working in the visible range between 550 and 564 nm and with a laser efficiency of 12%.

Ray et al. [77] finely tuned photophysical properties of stabilized N-BODIPYs (**28**, Scheme 2) thanks to the proper substitution of the fluorine linked to boron atom with electron-poor diamine moieties. Specifically, they demonstrated stabilized N-BODIPYs based on electron-poor amine moieties based on sulfonylated amines with both low flexibility and low steric hindrance, and electron-rich BODIPY cores were able to give rise to bright fluorophores even in the solid crystalline state, with notable lasing capacities in the liquid phase (surpassing 60% laser efficiencies) and when doped into polymers, improving the laser performance of their commercial counterpart PM567.

A series of BODIPYs with different substituted compounds was studied by Kuznetsova et al. In their work [78], they investigated how ligand structures and optical properties were related. They founded that alkyl-, cycloalkyl-, phenyl-, benzyl-, and meso-propargylamino substituted difluoroborates BODIPYs were photostable and laser active in liquid and solid-state in the spectral range between 475 and 687 nm. In particular, in solid-state, two derivatives, **29a** and **29b** (Scheme 2) with ethanyl and methyl groups (with and without phenyl group, respectively) showed very good performances: in PMMA they gained a laser efficiency up to 38% in PMMA and up to 58% in 8MMA-PMMA with a quite low threshold.

### 3.3. BODIPY as Active Layer in Optical Microcavity

**Figure 16:**
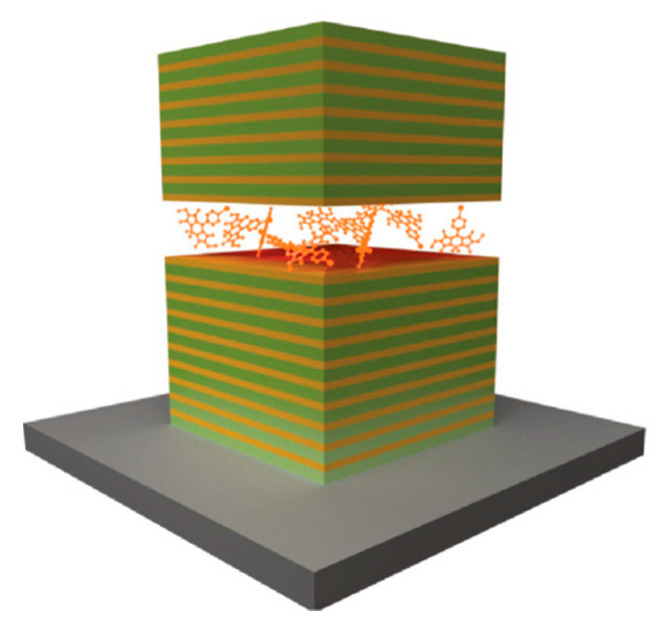
Schematic of the dye-filled microcavity. Reproduced from Ref. [105] with permission from 2017 WILEY-VCH Verlag GmbH & Co. KGaA, Weinheim, Germany.

In 2017, Cookson et al. [105] used BODIPY-Br (**30**, Scheme 2) to demonstrate polariton condensation in optical cavities. As shown in Figure 16, the dye was dispersed in a transparent polymeric matrix and then incorporated into a microcavity.

**Figure 17:**
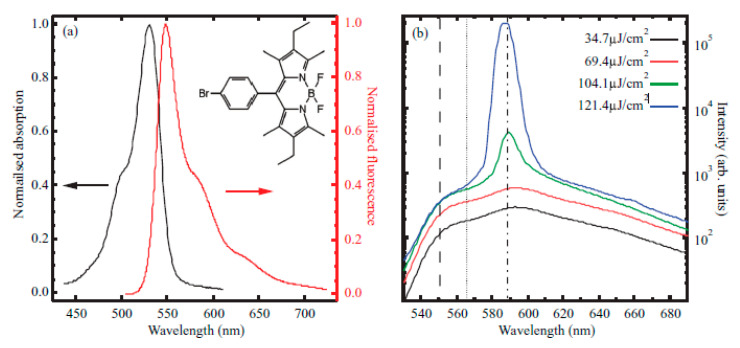
(**a**) The normalized absorption (black) and fluorescence spectrum (red) of BODIPY-Br dispersed in a transparent polystyrene matrix. The chemical structure is shown in the insert. (**b**) Amplified spontaneous emission from a 186 nm thick film of the BODIPY-Br (**39**, Scheme 2) dispersed in a polystyrene matrix and deposited on a quartz substrate. A threshold is observed at a pump fluence of 104 μJ cm^−2^ with a peak forming at 589 nm (dashed–dotted line). The dotted line indicates the polariton emission from the microcavity, while the large dashed line indicates the fluorescence emission peak shown in (**a**). Reproduced from Ref. [105] with permission from 2017 WILEY-VCH Verlag GmbH & Co. KGaA, Weinheim, Germany.

BODIPY-Br is characterized by relatively high photoluminescence (PL) quantum yield and optical amplification at 589 nm after pumping at 500 nm (see Figure 17). The work showed strong evidence of nonlinear PL with increasing excitation density, associated with a high linewidth narrowing and a continuous blueshift. This shift was assigned to polariton interactions with other polaritons and the exciton reservoir.

This work demonstrated the capability of achieving condensation at room temperature, in a dilute molecular system. This result opens the possibility to select polariton condensation wavelengths for the development of future optoelectronic devices, as hybrid organic-inorganic polariton laser diodes. There are many different molecular dyes that can be embedded in a microcavity to have polariton condensation at different wavelengths covering the visible and the NIR range.

**Figure 18:**
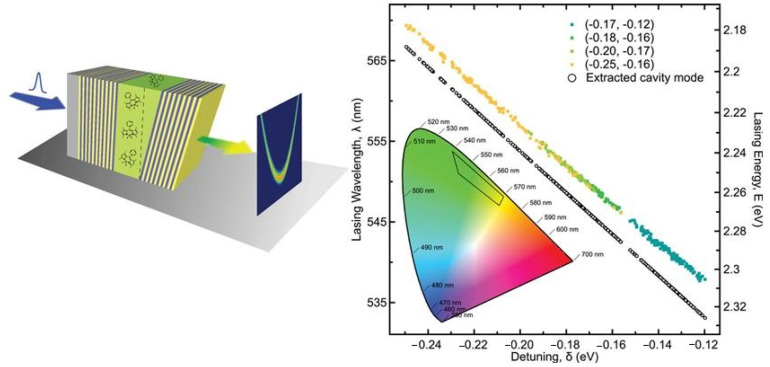
(**left**) Schematic representation of the wedged dye-filled microcavity and the excitation and detection configuration. (**right**) The colored solid-square data points indicate the wavelength (photon energy, right axis) where polariton lasing was observed versus the corresponding exciton–photon detuning. The black open circles indicate the corresponding bare cavity mode for each realization. Polariton lasing spans the green-yellow part of the visible spectrum as indicated with a black solid line on the CIE 1931 chromaticity diagram, bottom left inset. Reproduced from Ref. [106] with permission from 2019 WILEY-VCH Verlag GmbH & Co. KGaA, Weinheim, Germany.

In 2019, Sannikov et al. [106] developed a material system with polariton lasing at room temperature over a broad spectral range. The system is based on molecule BODIPY-G1 (Scheme 2), which is a derivative presenting high extinction coefficients and high PL quantum yield. The dye is dispersed in a polystyrene matrix and used as the active layer in a strongly coupled microcavity. The authors found that by engineering a thickness gradient across the microcavities (see Figure 18a), it was possible to gain a broad range of exciton–photon detuning conditions. As shown in Figure 18b, in this configuration, BODIPY-G1 microcavity can undergo polariton lasing over a broad range (around 33 nm) of wavelengths in the green-yellow spectral range, with a highly monochromatic emission line of 0.1 nm.

In conclusion, we have shown that a lot of work has been done on the achievement of lasing from BODIPY solutions or from “bulky” solid-state rods, but just in 2013 Durán-Sampedro [78] reported the use of a BODIPY thin film as an active layer in a photonic device as a Distributed Feedback Laser. Few other groups, since then, have implemented the use of thin films in photonic devices like DFB or microcavities lasers demonstrating the good properties of this material and its versatility.

## 4. Conclusions and Perspectives

The development of new organic semiconductor materials is certainly one of the pillars on which the possibility of bringing advanced technologies to the market and consequently making them accessible to all rests.

Since its discovery BODIPY molecule has shown great versatility thanks to the possibility of easy functionalization in different positions, fine modulation of optoelectronic properties, and good stability moreover it was possible its application in biochemical labeling chemosensors and fluorescent switches. Only recently BODIPY has emerged as effective building blocks for OPV and photonic applications and both these areas are indisputably at the basis of sustainable development.

In the last 5 years, essential improvements have been obtained in both applications thanks to the study of the effect of substituents and matrices for its dispersion.

This review explores the recent progress (2009–2020) in the field of solid-state BODIPY-based semiconducting molecules for photonics and OPVs applications. The review is divided into sections and provides a concise and precise description of the experimental results, their interpretation as well as the conclusions that can be drawn. After the first introductory section, in Section 2, we report the best results obtained in solar cells by focusing on devices with an efficiency greater than 5% and taking into consideration their use as an acceptor and as a donor molecule. In Section 3, we highlight the best results obtained in photonic applications such as lasing in DFB structures or microcavities. The chemical structure and the main optoelectronic properties, as well as their device performances and, where possible, their morphological properties are deeply investigated and summarized in Table 1, Table 2 and Table 3 evidencing that the most promising results were mostly achieved in the past 2–3 years.

Despite all advantages of using BODIPY-based chromophores, there are several challenges, which require more attention. For example, the effect of substituents on the Boron positions on the BODIPY properties has been only marginally investigated. These substitutions can allow to improve the stability of the material and to modulate the absorption and emission spectra towards the NIR spectral window. The development of hybrid systems with 2D materials could give a valuable contribution to the development of new multifunctional materials [107,108].

Finally, it is necessary to combine the sustainable aspect of the material, whose biocompatibility has already been demonstrated, with sustainable synthetic strategy and solubility in green solvents.

We hope that this review will provide a useful tool for the design and understanding of the structure–property relationship of BODIPY chromophores to facilitate their use in the optoelectronic research area.

## Figures and Tables

**Table 3:** Summary of laser parameters and dyes in the reported publications on bulk solid-state samples ^1^.

Molecule	Efficiency	λ_LASER_ [nm]	λ_EXC_ [nm]	Power [mJ]	Reference
**16**	up to 30%	559–564	534	5.5	[96]
**16**	up to 36%	561–564	532	5.5	[95]
**17**	up to 42%	576–579	532	5.5	[95]
**18**	25%	563	532	5.5	[97]
**19**	up to 48%	559–609	532	5	[98]
**20**	up to 45%	530–615	515 or 532	5	[99]
**21**	29%	522	355	5.5	[100]
**22**	up to 30%	565–615	532	5.5	[101]
**23**	56%	568	532	5	[102]
**24**	53%	588	532	5	[103]

^1^ Efficiency: energy conversion efficiency. λ_LASER_: peak of the laser emission. λ_EXC_: pump excitation (transversal). Power: pump power/pulse**_._**

**Table 4:** Summary of laser parameters and dyes in the reported publications on thin films ^1^.

Molecule	λ_LASER_ [nm]	λ_EXC_ [nm]	Threshold [MW/cm^2^]	Reference
**23**	570	532	1.7 × 10^−3^	[78]
**24**	588	532	20 × 10^−3^	[101]
**25**	680	580	0.34	[102]
**26**	562–566	532	3	[103]
**27**	557	532	10–20	[104]
**28**	569	532	17 × 10^−3^	[105]
**29**	562–566	532	3	[106]

^1^ λ_LASER_: peak of the laser emission; λ_EXC_: pump excitation.

## Abbreviations

BDT	Benzo[1,2-b:4,5-b’]dithiophene
BHJ	Bulk-HeteroJunction solar cells
BPAPF	9,9-bis[4-(*N*,*N*-bis-biphenyl-4-yl-amino)phenyl]-9H-fluorene
D–A	Donor–Acceptor
DCM	Dichloromethane
DFB	Distributed Feedback laser
DPP	Diketopyrrolopyrrole
ETL	Electron Transporting Layer (ETL)
FF	Fill Factor
HOMO	Highest Occupied Molecular Orbital
HTL	Hole Transporting Layer
ITO	Indium Tin Oxide
Jsc	Short-circuit current Density
LUMO	Lowest Unoccupied Molecular Orbital
MH250	*N*,*N*-bis(fluoren-2-yl)-naphthalenetetracarboxylic diimide(bis-Hfl-NTCDI)
MMA	Methyl Methacrylate
MoO_3_	Molibdenum Oxide
NDP9	Organic p-type dopant of *Novaled* GmbH
NIR	Near InfraRed
OPV	Organic PhotoVoltaic
OSC	Organic Solar Cell
P3HT	Poly(3-hexylthiophene-2,5-diyl)
PAZO-Na	Poly[1-[4-(3-carboxy-4-hydroxyphenylazo)benzenesulfonamido]-1,2-Ethanediyl, sodium salt]
PCE	Power Conversion Efficiency
p-DTS-(FBTTh_2_)_2_	7,7′-[4,4-Bis(2-ethylhexyl)-4*H*-silolo[3,2-b:4,5-b′]dithiophene-2,6-diyl]bis[6-Fluoro-4-(5′-hexyl-[2,2′-bithiophen]-5-yl)benzo[c][1,2,5]thiadiazole]
PEDOT:PSS	Poly(3,4-ethylenedioxythiophene) polystyrene sulfonate
PEIE	Polyethylenimine ethoxylated
PFN	Poly[9,9-bis(3′-(*N*,*N*-dimethylamino)propyl)-2,7-fluorene
PL	Photoluminescence
PMMA	Poly(methyl methacrylate)
PTB7-Th	Poly([2,6′-4,8-di(5-ethylhexylthienyl)benzo[1,2-b;3,3-b]dithiophene]{3-fluoro-2[(2-ethylhexyl)carbonyl]thieno[3,4-b]thiophenediyl})
SCLC	Space-Charge Limited-Current
SVA	Solvent Vapor Annealing
TA	Thermal Annealing
TAT	Triazatruxene
TF	Thin film
TFMA	2-trifluoromethyl methacrylate
TSVA	Thermal and Solvent Annealing
V_oc_	Open Circuit Voltage
W_2_(hpp)_4_	Tetrakis(1,3,4,6,7,8-hexahydro-2*H*-pyrimido[1,2-a]pyrimidinato)ditungsten (II)
ZnO	Zinc Oxide
